# Axially lattice-matched wurtzite/rock-salt GaAs/Pb_1−*x*_Sn_*x*_Te nanowires

**DOI:** 10.1038/s41598-024-51200-w

**Published:** 2024-01-05

**Authors:** Sania Dad, Piotr Dziawa, Wiktoria Zajkowska-Pietrzak, Sławomir Kret, Mirosław Kozłowski, Maciej Wójcik, Janusz Sadowski

**Affiliations:** 1 Institute of Physics, Polish Academy of Sciences, Aleja Lotnikow 32/46, 02-668 Warsaw, Poland; 2 Faculty of Physics, University of Warsaw, Pasteura 5, 02093 Warsaw, Poland; 3 Ensemble3 Centre of Excellence, Wolczynska Str. 133, 01-919 Warsaw, Poland

**Keywords:** Materials science, Nanoscience and technology, Physics

## Abstract

We investigate the full and half-shells of Pb_1−*x*_Sn_*x*_Te topological crystalline insulator deposited by molecular beam epitaxy on the sidewalls of wurtzite GaAs nanowires (NWs). Due to the distinct orientation of the IV–VI shell with respect to the III–V core the lattice mismatch between both materials along the nanowire axis is less than 4%. The Pb_1−*x*_Sn_*x*_Te solid solution is chosen due to the topological crystalline insulator properties above some critical concentrations of Sn (*x* ≥ 0.36). The IV–VI shells are grown with different compositions spanning from binary SnTe, through Pb_1−*x*_Sn_*x*_Te with decreasing *x* value down to binary PbTe (*x* = 0). The samples are analysed by scanning transmission electron microscopy, which reveals the presence of (110) or (100) oriented binary PbTe and (100) Pb_1−*x*_Sn_*x*_Te on the sidewalls of wurtzite GaAs NWs.

## Introduction

The combination of semiconductor heteroepitaxial systems without the generation of numerous defects often remains inaccessible due to the large lattice mismatch between materials of interest^1–3^. However, in materials stacked in nanostructures the lattice matching requirements indispensable in the case of large area planar heterostructures are much less severe. In the nanoscale dimensions, the stress/strain can be shared between both components of the heterostructures, in contrast to the case of thin layers grown on substrates usually thicker by several orders of magnitude^4,5^. In this report, we present a successful attempt of combining two much dissimilar materials (with the lattice misfit from 12% to 14%) in the core–shell nanowire heterostructures, namely well-known III–V semiconductor GaAs in the wurtzite (WZ) crystalline phase (occurring in the nanowires only) and a narrow bandgap IV–VI semiconductor (Pb_1−*x*_Sn_*x*_Te solid solution) exhibiting also topological crystalline insulator (TCI) properties.

In the last decade, Pb_1−*x*_Sn_*x*_Te was identified as a TCI with protected gapless electronic states on its high symmetry surfaces or edges^6^. Topological surface states (TSS) are protected by crystal symmetry in contrast to the time-reversal symmetry protection in formerly known Z_2_ topological insulators (TIs)^6,7^. Apart from the topological properties, narrow bandgap IV–VI semiconductors possess other unique features making them promising materials for applications in thermoelectric and mid-infrared optoelectronic devices such as photodetectors, thermoelectric coolers, infrared light sources (lasers), and detectors^8,9^.

Investigations of the IV–VI narrow bandgap lead–tin chalcogenides have gained momentum over the last decade due to the discovery of TSS on high-symmetry surfaces. The indispensable conditions for the occurrence of TSS, namely mirror symmetry of the crystalline lattice and electronic band inversion are fulfilled in certain IV–VI compounds^10^. Shortly after the first theoretical report on the TCI^11^, the topological phase protected by the crystalline symmetry has been theoretically predicted^12^ and confirmed experimentally for binary SnTe^13^, Pb_1−*x*_Sn_*x*_Te^14^, and Pb_1−*x*_Sn_*x*_Se^15^ solid solutions. To maximize the effects related to the topologically protected states^16,17^ the quasi-one-dimensional nanowires (NWs) are beneficial due to their high surface-to-volume ratio. Moreover, NWs enable explorations of novel phenomena such as topologically protected 1D hinge states emerging along the NW edges^18^. Several surfaces hosting topologically protected states in individual NWs—four surfaces with topologically protected states at the NW sidewall surfaces of native IV–VI NWs with square cross-section^18^ and six such surfaces for GaAs-(IV–VI) core–shell NWs with hexagonal cross-section^19^ described here, can occur in the NWs.

Over two decades of research on semiconducting NWs paved way for the fabrication of highly controllable NW assemblies, in terms of their composition, lengths, diameters, ordered positioning, and crystal structure^20–25^. One of the basic methods of the NWs growth exploits the vapour-liquid-solid (VLS) mechanism identified by Wagner and Ellis half a century ago^26^. This technique is still an excellent approach to obtain high-quality NWs^21–24,27,28^. A typical VLS mechanism requires a liquid nanodroplet (catalyst), defining distinct NW diameters. The VLS approach has been successfully applied to obtain NWs of II–VI, III–V, Si, and IV–VI semiconductor NWs^29–31^.

In comparison to simple NWs, the NW heterostructures offer numerous new functionalities^32–37^, especially the core–shell NWs enable a wide range of applications due to their capabilities for altering the material properties. For example, the band gap of the core can be tuned by straining the nanowire using the lattice mismatched shell^38^. Auger recombination and surface trap states are reduced in the core–shell NW structures, the appropriately designed shells can also improve the optical and charge transport properties of the core material^32^. Carrier multiplication^39^ phenomenon can lead to promising performances in photodetectors, solar cells, and light-emitting diodes^40,41^. Recently, PbTe–PbS 1D core–shell nanostructures were reported, in which transport of electrons was prevailing over the ambipolar one. Such a property makes them a suitable candidate for thermoelectricity and photovoltaic devices^42,43^. IV–VI materials are usually grown on potassium chloride or barium fluoride substrates which, however, results in poor mechanical stability, unfavourable thermal performance, or difficulties in processing due to the reactivity of such substrates with water. Therefore, the growth of IV–VI (rock-salt structure) on Si and III–V or II–VI (zinc-blende structure) substrates is preferred. This approach offers several merits such as better surface morphology and crystalline perfection^44–46^. Moreover, the substantial lattice mismatch of IV–VI materials to Si or GaAs substrates is not an obstacle for the NWs growth due to the small lateral NW dimensions. Due to the same reasons the reduced dislocation densities at interfaces can be obtained in heterostructures with lattice mismatched core–shell NWs^47^. In our WZ GaAs-(Pb,Sn)Te core–shell NW heterostructures we get use of the relatively good lattice matching between the (0001) lattice planes of WZ GaAs (interplanar spacing of 6.570 Å) and (001) ones of the rock-salt Pb_1−*x*_Sn_*x*_Te (interplanar spacing ranging from 6.46 Å to 6.32 Å for *x* = 0 to *x* = 1, respectively). This results in a low lattice mismatch (of 1.7% up to 4%) along the NW axis. The lattice mismatch along the sidewalls in the direction perpendicular to the NW axis is much higher, but since the lengths of the NWs are one order of magnitude bigger than the lateral dimensions of the NW sidewall we do expect a reduced number of misfit dislocations in both directions. Such a situation does not occur in layered heterostructures implementing highly mismatched III–V and IV–VI semiconductors, due to the lack of a shape anisotropy characteristic for NWs. Usually, a high density of misfit dislocations and/or microcracks was observed for IV–VI materials grown directly on ZB GaAs^46,48^ even with the use of a CdTe buffer layer with a very good lattice match to IV–VI compounds^49^. Inspired by good lattice matching to WZ GaAs we grow and investigate Pb_1−*x*_Sn_*x*_Te, both shells and half-shells, deposited on the sidewalls of WZ GaAs NW cores. Since the sidewalls of WZ GaAs NWs are constituted from $\{1\overline{1}00\}$, or $\{11\overline{2}0\}$ surfaces, we expect to obtain {110} or {100} surfaces of the IV–VI shells, respectively, provided that epitaxial relation between the III–V core and the IV–VI shell material is maintained. Moreover, in the further research perspective we head for investigations of the theoretically predicted one-dimensional higher-order topological insulator (HOTI) states at the NW edges^18^. In such a hybrid (III–V)/(IV–VI) configuration it is possible to obtain relatively long NWs, contrary to the typical MBE-grown IV–VI NWs^27,50^, which is beneficial for studying their characteristics involving topologically protected electronic states^51^.

The core–shell NWs are examined by scanning electron microscopy (SEM) and scanning transmission electron microscopy (STEM). The lattice mismatches of the shells with respect to the core are measured based on STEM images and compared with the theoretical calculations. To our knowledge, Pb_1−*x*_Sn_*x*_Te shells deposited on GaAs NW cores have not been reported before.

## Results and discussion

**Figure 1:**
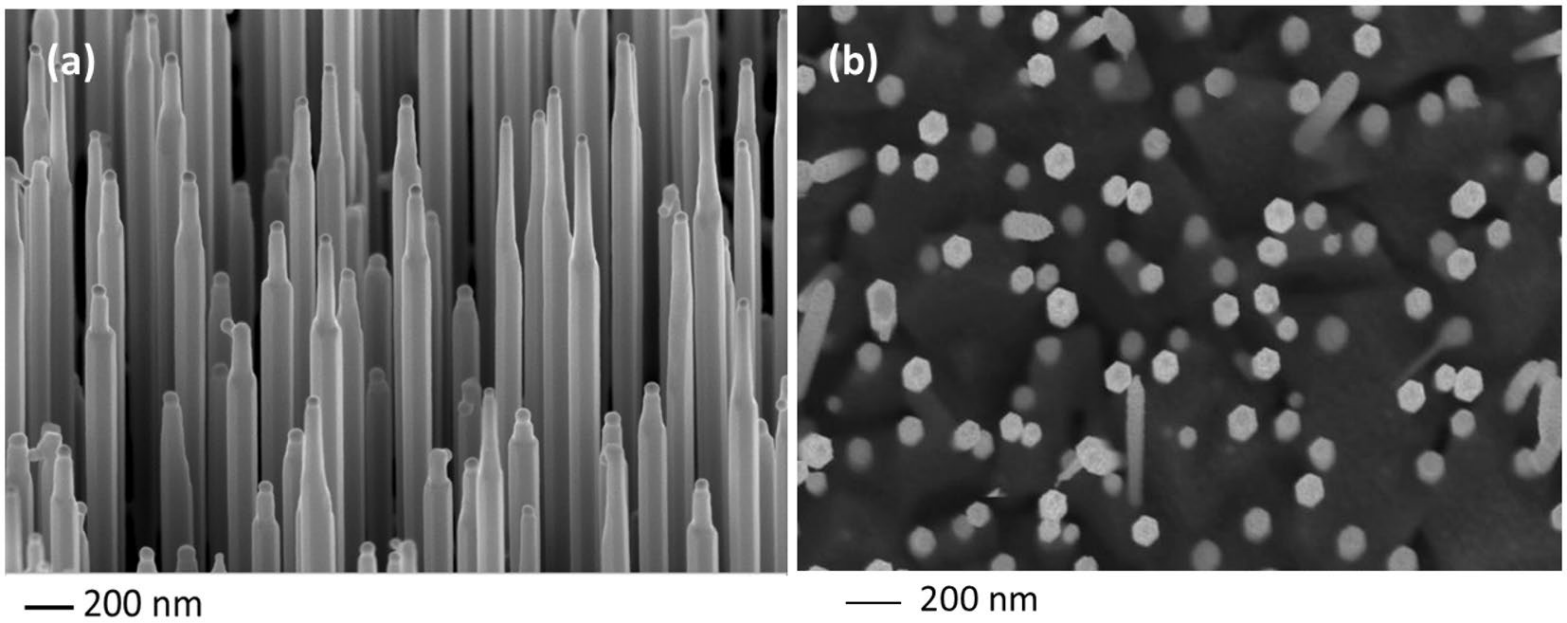
The side (**a**) and top (**b**) view of WZ GaAs NWs grown by gold-assisted VLS method.

The Au-catalyzed GaAs NWs are grown on GaAs (111)B substrates in the III–V MBE system. The NWs have been grown in conditions favouring wurtzite (WZ) crystal structure and their axes follow the [0001] direction (see SEM images in Fig. 1). Then, III–V NWs were transferred (in the air) to the separate IV–VI MBE system and used as templates (cores) for IV–VI shells deposition. GaAs NWs were annealed to thermally desorb the native oxides from the NW sidewalls. After annealing, the substrate temperature was decreased down to the temperature favourable for the growth of IV–VI shells. Pb_1−*x*_Sn_*x*_Te shells with various chemical compositions are grown with *x* ranging from 0 to 1.

GaAs NWs used as cores for the growth of Pb_1−*x*_Sn_*x*_Te shells is undoped with the Fermi level in the middle of the energy gap (E_g_ = 1.42 eV for ZB GaAs at room temperature). WZ GaAs is also a direct bandgap semiconductor with the energy gap very close to that of ZB GaAs^52^. Pb_1−*x*_Sn_*x*_Te is typically a p-type narrow bandgap semiconductor (*E*_g_ ≤ 0.32 eV at room temperature) with the Fermi level in the valence band. Minimum of the band-gap in GaAs is placed at the  Γ point of the Brillouin zone (BZ), and in the case of IV–VI compounds the minimum of the energy gap under normal conditions is located at the L point of BZ. Hence, GaAs constitutes a large barrier for the Pb_1−*x*_Sn_*x*_Te valence band holes and there is no charge transfer between the core and the shell.

In order to keep our core–shell NWs suitable for transmission electron microscopy investigations (transparent to e-beam) we have chosen relatively low thicknesses of IV–VI shells of about 10 nm which is equivalent to about 30 monolayers (ML). One can argue if such relatively low thickness does not lead to the hybridization of the wave functions of surface states at inner and outer shell surfaces. The penetration depth of topological surface states depends on the material. In the case of IV–VI TCI, the decay is usually expected to be less than c.a. 10 nm deep into the material. Theoretical predictions show vanishing TCI state below few monolayers, where 1 ML ≈ 0.3 nm^53^. However, TCI is expected to be observed again for a layer thickness close to 1 ML^54^. Experimental proof of the critical distance is presented for 1D TI states on the surface of (Pb,Sn)Se. Using scanning tunnelling spectroscopy it was shown that edge states disappear at a distance less than c.a. 10 nm^55^. To prevent cancelation of closed-gap TCI states thick enough shells should be used.

### MBE growth and structural properties of IV–VI shells

#### SnTe shells

**Figure 2:**
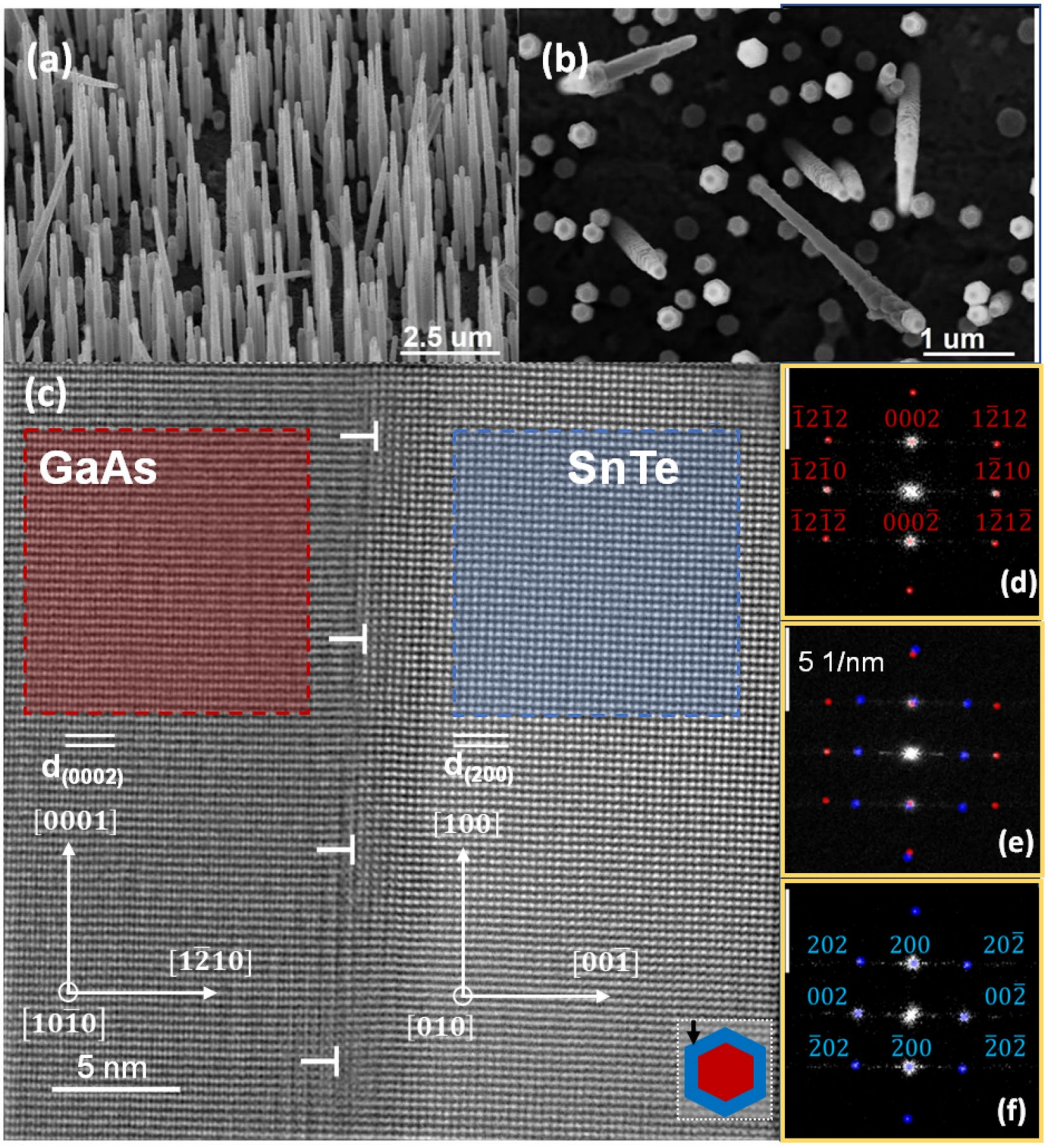
(**a**,**b**) SEM images (side and top view) of WZ GaAs/SnTe core–shell NW at a magnification of 10 k and 25 k, respectively. (**c**) STEM image of GaAs $(1\overline{2}10)$/SnTe(100) interface along the [010] projection of SnTe, misfit dislocations in the shells at the interface with the core region are marked with “T” (also in all subsequent Figures with TEM images); (**d**–**f**) indexed 2D fast Fourier transforms (2D FFT) of images; red and blue dots correspond to GaAs and SnTe, respectively; the FFT patterns share the same scale of 5 nm^−1^ (bars at the top-left). The bottom-right inset to the panel (**c**) depicts hexagonal cross-sectional shape of the NW and its orientation with respect to the electron beam (marked as a black arrow) used in TEM.

The SEM and TEM images of WZ GaAs NWs with SnTe shells are presented in Fig. 2. The SnTe shells are quite well lattice matched to the WZ GaAs NW core in the axial direction due to the specific orientation of both crystalline lattices as explained below. Figure 2c shows the high-resolution scanning transmission electron microscopy (STEM) image of the WZ GaAs/SnTe interface revealing the alignment of the SnTe shell and WZ GaAs NW core crystal lattices. Corresponding electron diffraction image indexings are depicted in Fig. 2d–f. The image of the GaAs/SnTe interface displayed in Fig. 2c is taken in the GaAs[$10\overline{1}0]$ zone axis.

We define the misfit parameter f as:

1 $$f=({d}_{{\text{sub}}}-{d}_{{\text{lay}}})/{d}_{{\text{lay}}}$$

where ${d}_{sub}$ and ${d}_{lay}$ are the interplanar spacings in the substrate and the layer material, respectively. The values of the interplanar spacings along the specific directions obtained from the STEM analysis are calculated using the following lattice parameters: $c=6.57$ Å and $a=3.9845$ Å for WZ GaAs^56^, and $a=6.318$ Å for SnTe^57^. These give the distances *d* between WZ GaAs and SnTe lattice planes: ${d}_{(0002)}^{{\text{GaAs}}}=3.285$ Å and ${d}_{(200)}^{{\text{SnTe}}}=3.159$ Å.

Based on the interplanar distances we define the accumulated strain as:

2 $$\varepsilon =({d}_{{\text{theor}}}-{d}_{{\text{exp}}})/{d}_{{\text{theor}}}$$

where ${d}_{{\text{theor}}}$ is a theoretical value of an interplanar distance and ${d}_{{\text{exp}}}$ is the experimental one.

Here we neglect the strain exerted by IV–VI shell on WZ GaAs NW core; i.e., we treat WZ GaAs lattice parameter values as a “frame of reference”. Indeed, as shown by Ferrand and Cibert^58^ even in GaN-AlN core–shell NWs—materials much stiffer than SnTe and GaAs, the strain in the core is below 0.5% for the ratios of core to shell radii similar to our case. The mismatch between the spacing of (0002) planes of WZ GaAs and the (002) ones of SnTe along the NW axis amounts to ${f}_{{\text{theor}}\,[0002]}^{{\text{SnTe}}/{\text{GaAs}}}=0.03989$.

We can calculate the actual lattice mismatch along the NW axis, ${f}_{{\text{exp}}}^{{\text{SnTe}}/{\text{GaAs}}}$, on the basis of the high-resolution TEM (HR-TEM) images. Considering the fact that the core is much thicker than the shell, we assume the inter-planar spacing value for GaAs to be ${d}_{(0002)}^{{\text{GaAs}}}=3.285$ Å. The inter-planar spacing of SnTe shell measured from the image displayed in Fig. 2c is ${d}_{(200)}^{{\text{SnTe}}}=3.17$ Å. Using this measured value, the difference in the interplanar distances between the core and the shell according to Eq. (1) is ${f}_{{\text{exp}}\,[0002]}^{{\text{SnTe}}/{\text{GaAs}}}=0.0363$. This value is 10% smaller than the theoretical one which indicates that the SnTe shell is partially strained (elongated) along the NW axis with the strain value of ${\varepsilon }_{002}^{{\text{SnTe}}}\cong$ 0.348%, following the Eq. (2).

#### Pb_0.65_Sn_0.35_Te shells

**Figure 3:**
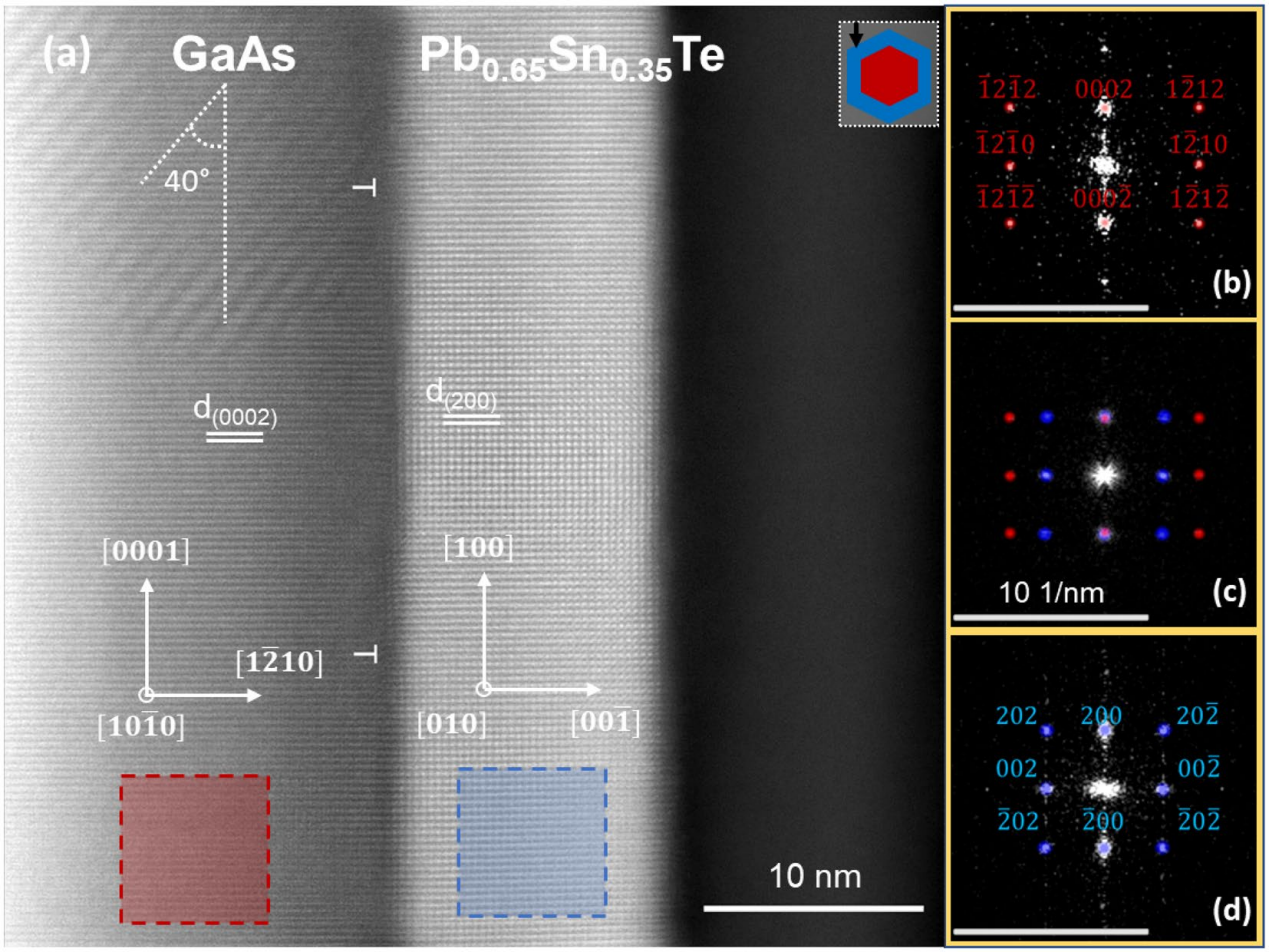
(**a**) STEM image of GaAs $(\overline{1}2\overline{1}0)$/Pb_0.65_Sn_0.35_Te(100) interface along the [010] projection of the shell. 2D FFT patterns of the core (**b**), interface (**c**), and the shell (**d**) are presented in the panels with yellow frames, where red dots correspond to the core and blue ones to the shell, sharing the same scale of 10 nm^−1^ (bars at bottom-left). The inclined fringes in the upper middle panel are due to the moiré effect of (Pb,Sn)Te grain parallel to the image plane and rotated 40 degrees with respect to the NW axis. The upper right inset to the panel (**a**) depicts the orientation of the NW with respect to the electron beam (marked as a black arrow) used in TEM.

Another type of the IV–VI shells on WZ GaAs NW cores studied here constitutes from Pb_1−*x*_Sn_*x*_Te solid solution with Sn content *x* = 0.35^59,60^. For such a concentration of Sn the topological phase transition can occur in Pb_1−*x*_Sn_*x*_Te at low temperatures^61^. The high magnification picture of the core–shell structures and their interface along the [1010] projection of GaAs NW is shown in Fig. 3a.

The well-visible fringes at the upper part of the NW core (GaAs) are recognized as the moiré patterns. Two main causes of moiré fringes are translational and rotational contributions due to the difference in the lattice planes spacings of the core and the shell fragments, and possible rotation between these lattices (also see S.2. in Supplementary information). In Fig. 3a, a small area with rotational moiré fringes is visible (see top left). The whole shell grain is twisted by 40° (marked with white broken lines). The right panels in Fig. 3b–d show the FFT images of the core, core–shell, and the shell respectively. For calculation of the lattice mismatch, the lattice parameter of Pb_1−*x*_Sn_*x*_Te for *x* = 0.35 is taken as *a* = 6.4116 Å at room temperature (calculated according to the Vegard’s law)^62^. The interplanar spacing (*d*) values for GaAs (0002) and Pb_1−*x*_Sn_*x*_Te (200) are calculated as 3.285 Å and 3.206 Å respectively. The lattice misfit due to the difference in the lattice parameters of the core and the shell calculated using the same expression given in Eq. (1) ${f}_{{\text{theor}}\,[0002]}^{{\text{PbSnTe}}/{\text{GaAs}}}=0.02464.$ The interplanar spacing of Pb_0.65_Sn_0.35_Te shell measured from the high-resolution STEM image ${d}_{(200)}^{{\text{PbSnTe}}}=3.22$ Å. Based on these values we calculated misfit ${f}_{{\text{exp}}\,[0002]}^{{\text{PbSnTe}}/{\text{GaAs}}}=0.0202,$ which is smaller than the theoretical value. Hence the actual misfit is lower and according to Eq. (2) Pb_0.65_Sn_0.35_Te is strained along the axial direction by ${\varepsilon }_{200}^{{\text{PbSnTe}}}\cong$ 0.437%.

#### PbTe shells

**Figure 4:**
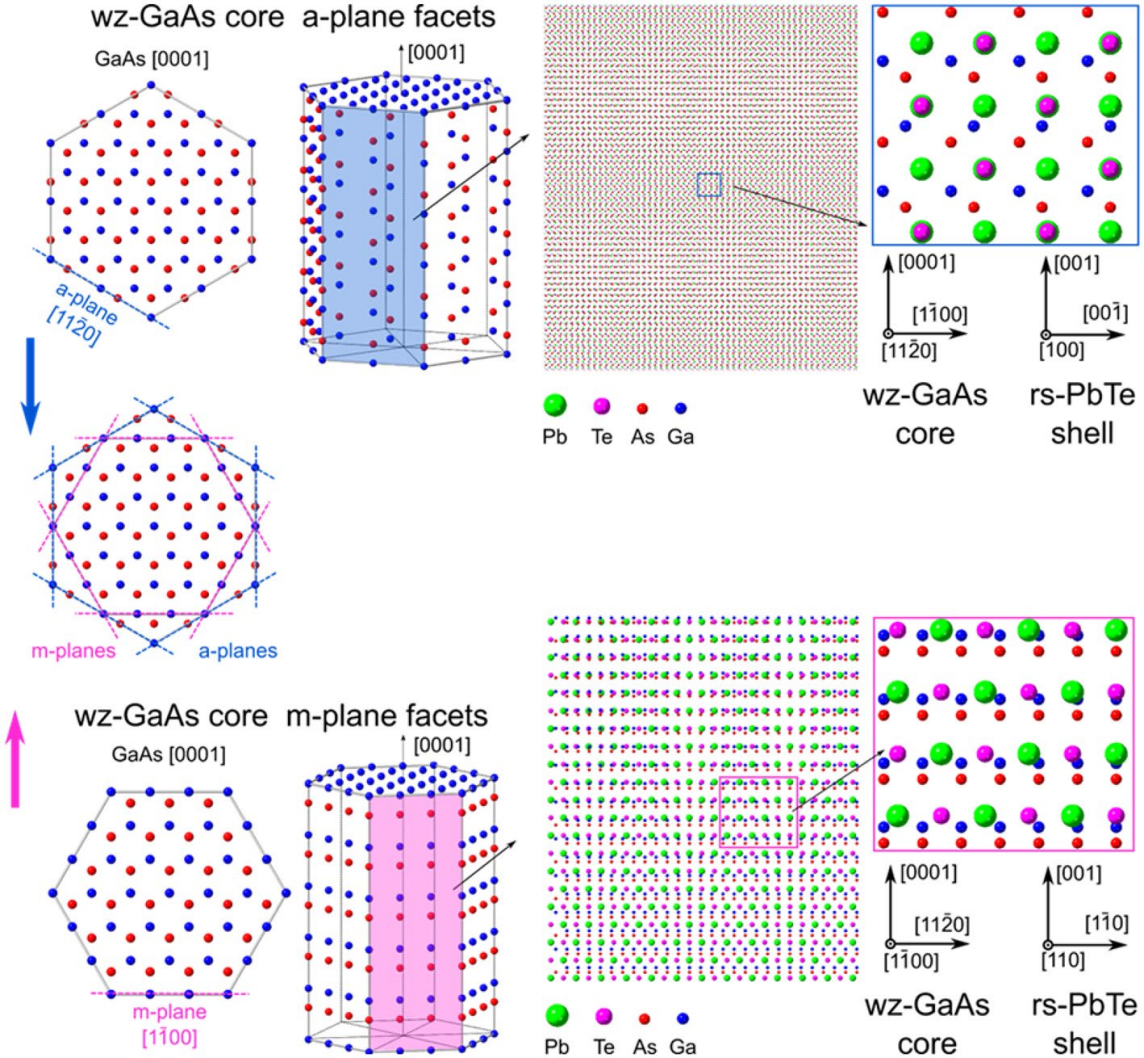
Schematic representation of the WZ GaAs nanowire with two types of sidewall facets (made using Crystal Maker software). Upper panel: $\{11\overline{2}0\}$ WZ GaAs sidewalls (a-planes); lower panel $\{1\overline{1}00\}$ sidewalls (m-planes). The left panel of the figure is a pictorial illustration of top and side view of WZ GaAs nanowire together with an indication of a-plane with blue colour (top left) and m-plane with pink colour (bottom left). The right panel of the scheme depicts lower and higher magnification of m- and a-plane facets, with (100) orientation of rock-salt PbTe shell on a-plane and (110) rock-salt PbTe shell on m-plane, respectively.

In WZ GaAs NWs two types of the sidewall facets can occur: $\{\overline{1}100\}$ and $\{11\overline{2}0\}$, m- and a-planes, respectively. The left side of Fig. 4 presents the differences between m- and a-plane facets of WZ NW. We have observed two distinct orientations of PbTe shells depending on which type of WZ GaAs core facet they are deposited. PbTe shells grow with {100} orientation on the a-plane facets, whereas {110} shell orientation is evidenced on the m-plane ones. In both GaAs/PbTe orientations: $\{11\overline{2}0\}$||{100} (upper panels of Fig. 4) and $\{\overline{1}100\}$||{110} (lower panels in Fig. 4), view of atoms arrangements along the core–shell NW side wall is presented. The graphics corresponding to low magnification view evidence possible moiré patterns which can appear in TEM images. The zoomed areas of the visualisations are shown in the right panels. The simple geometrical analysis of these two situations shows that indeed such a matching is preferred. There are repeated areas of overlapping and non congruent atoms of the core and the shell. The good matching corresponds to the configuration with higher density of overlapping core and shell atoms in low magnification visualisations. On the other hand, the areas where shell and core atoms do not overlap in the view along the sidewall, correspond to a local mismatch of both crystal lattices. In the proposed core–shell orientation models, matching between two distinct facets of the WZ GaAs core and two orientations of the rock-salt PbTe shell observed by us is the best. In the samples investigated by us the individual GaAs core NW has a-type or m-type sidewall facets (not mixed). We don’t analyse here the reasons for formation of given type of facets in WZ GaAs NWs. Most likely {1–100} WZ facets are promoted in NWs with small diameters. The detailed explanation would need more studies of formation of WZ GaAs NWs in the III–V MBE growth chamber and goes beyond the scope of this paper. The possibility to grow PbTe(110) on the m-plane facets is especially interesting. The growth of PbTe, SnTe, and (Pb,Sn)Te in this orientation has not been reported, to our knowledge. Additionally, the (110) surface plane is the last of three surfaces of IV–VI TCI for which theoretically predicted Dirac surface states have not yet been experimentally observed.

**Figure 5:**
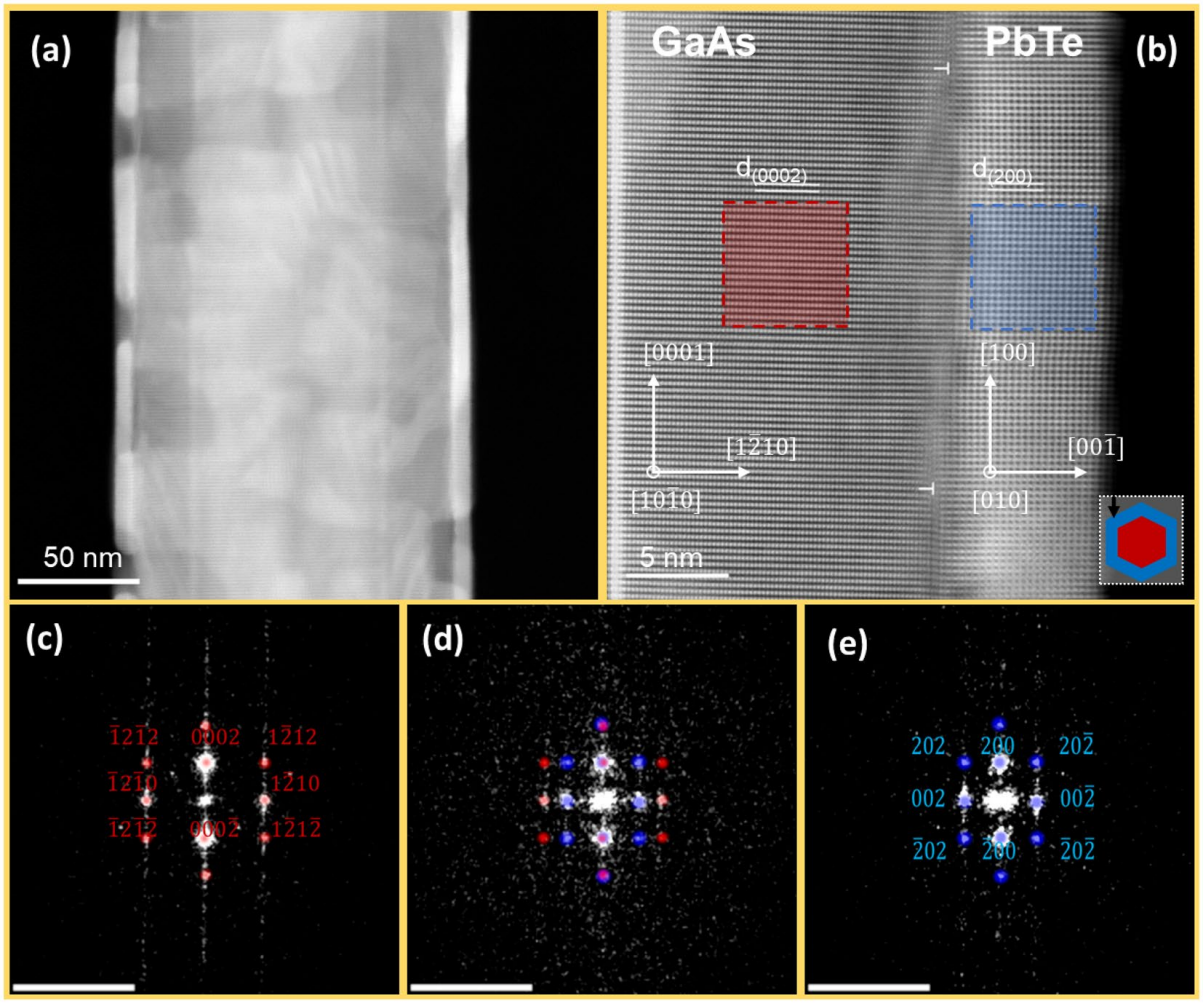
TEM images of (001) PbTe shells on WZ GaAs NW with $\{1\overline{2}10\}$ sidewalls: (**a**) individual core–shell NW with locally smooth but non-continuous PbTe shell with side-wall facet perpendicular to the e-beam, (**b**) high resolution image of a smooth core–shell interface along the [010] projection of PbTe. Lower panels show indexed 2D FFT patterns of (**c**) GaAs, (**d**) GaAs/PbTe interface, and (**e**) PbTe and white bar at the bottom of each image indicates the scale bar of 10 nm^-1^. Red colour in the figure panels corresponds to the core and blue one to the shell. The bottom-right inset to the panel (**b**) depicts the orientation of the NW with respect to the electron beam (marked as a black arrow) used in TEM.

Figure 5 shows TEM images of GaAs NW with $\{11\overline{2}0\}$ sidewall facets and {001} PbTe shells grown on them. A low magnification image (see Fig. 5a) evidences that the PbTe shell is very smooth. Some degree of discontinuity in the shell most probably occurs because of the low shell thickness (about 5 nm) and nonperfect coalescence of the initial growth islands at this low shell thickness limit. The variable contrast areas visible in the PbTe NW shell in about 230 nm long GaAs-PbTe core shell NW section are due to the incomplete coverage of the core NW by discontinuous PbTe islands. Darker areas represent gaps in the shells, as schematically depicted in Fig S5 of the Supplementary information. Figures 5(c-e) show the indexed electron diffraction images indicated by red and blue dots corresponding to the core and the shell, respectively. Along the [0001] GaAs NW axis the lattice matching between the GaAs core and PbTe shell is the best, as compared to SnTe and Pb_0.65_Sn_0.35_Te shells. The spacing of (200) PbTe planes is ${d}_{\left(200\right)\,{\text{theor}}}^{{\text{PbTe}}}=3.23$ Å, and thus ${f}_{{\text{theor}}\,[0002]}^{{\text{PbTe}}/{\text{GaAs}}}=0.01703$. At such a low lattice mismatch, less than 2%, the occurrence of misfit related defects are expected to be almost negligible. This can be seen in HR-TEM images (Fig. 5b). The spacing of PbTe (200) planes measured from the TEM images is ${d}_{\left(200\right)}^{{\text{PbTe}}}=3.25$ Å and corresponding lattice mismatch between WZ GaAs core and PbTe shell measured along the axial direction is ${f}_{{\text{exp}}\,[0002]}^{{\text{PbTe}}/{\text{GaAs}}}=$ 0.0108.

Along the [$1\overline{1}00]$ direction of GaAs NW sidewall, i.e. direction perpendicular to the NW axis and parallel to the sidewall plane, which we will call “tangential direction*”* the lattice matching between the core and the shell is substantially worse, but since the width of the shell is small about 100 nm. This misfit is much less important than the one along the axis of several micrometres long NW core. For the $\{11\overline{2}0\}$ WZ GaAs NW facet (a-plane) the corresponding interlayer distances (the theoretical bulk crystal values) amount to ${d}_{(1\overline{1 }00)}^\text{GaAs}=3.4507$ Å and ${d}_{(200)}^\text{PbTe}=3.23$ Å and the resulting theoretical mismatch for this orientation is ${f}_{\text{theor}\,[1\overline{1 }00]}^\text{PbTe/GaAs}=0.068.$ One can see that the misfit between the PbTe shell and WZ GaAs $\{11\overline{2}0\}$ facet in the tangential direction is over three times larger than in the axial one.

**Figure 6:**
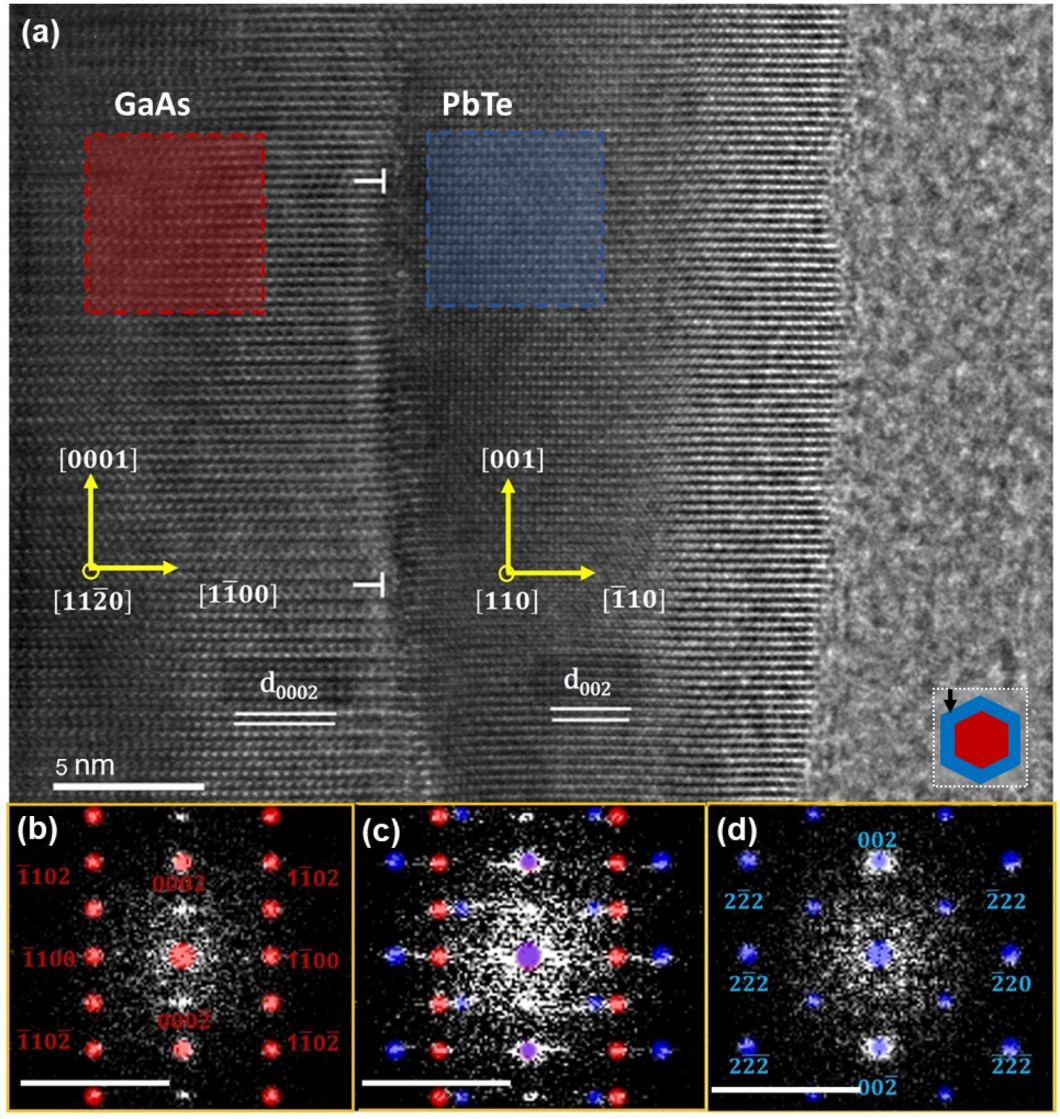
TEM images of ($\overline{1 }$10) PbTe shell on WZ GaAs nanowire with $\{1\overline{1}00\}$ sidewalls: (**a**) high-resolution image of the core–shell interface, (**b**–**d**) indexed 2D FFT patterns of GaAs, GaAs/PbTe interface region, and PbTe; the white bar at the bottom of each FFT image indicates the scale bar of 10 nm^−1^. Red and blue colour of spots on 2D FFT images correspond to the core and the shell, respectively. The bottom-right inset to the panel (**a**) depicts the orientation of the NW with respect to the electron beam (marked as a black arrow) used in TEM.

Figure 6 shows TEM images (along the $[11\overline{2}0]$ projection) of the GaAs core NW with $\{1\overline{1}00\}$ WZ GaAs m-plane facet and PbTe shell deposited on it. On $\{1\overline{1}00\}$ sidewall GaAs NW facets PbTe shells grow in {110} orientation. Here the lattice mismatch in the axial direction is the same as in the case of PbTe shells on $\{11\overline{2}0\}$ sidewall facets, but in the tangential direction, the (110) PbTe shell is much better matched to WZ GaAs, than the (100) one. For the $\{1\overline{1}00\}$ WZ GaAs NW facet (m-plane) the corresponding interlayer distances (the theoretical bulk crystal values) and calculated tangential mismatch (between the lattice planes perpendicular to the sidewall and parallel to the NW axis) are ${d}_{(11\overline{2 }0)}^\text{GaAs}=1.9922$ Å and ${d}_{(220)}^\text{PbTe}=2.2839$ Å, giving a mismatch value of ${f}_{\text{theor}\,[11\overline{2 }0]}^\text{PbTe/GaAs}=-0.1277.$ The mismatch to the {200} PbTe planes with the interplanar distances of 3.23 Å would be much higher, equal to −0.383. Hence the {110} orientation of the PbTe shells is much more favourable on $\{1\overline{1}00\}$ WZ GaAs NW facets. This is indeed observed in our samples (see Fig. 6).

The descriptive summary of all the calculated and measured values of the axial lattice mismatches and the dislocation distances for both binary PbTe and SnTe, and ternary Pb_1−*x*_Sn_*x*_Te compounds are given in Table 1. The theoretical distances between dislocations are calculated by simple considerations of coincidence site lattice. The main consequence of dislocations is releasing the stress in the overgrown structure which influences the electronic structure of a material. Theoretical predictions and experimental indications show that closed-gap TCI state can open at a high enough value of pressure^63,64^. The dislocation distance (marked with a ‘T’ sign in the each HR-TEM figure) increases with decreasing Sn content. This is due to the fact that the lattice mismatch between the core and the shell decreases resulting in the less relaxed shell lattice structure.

**Table 1:** Experimental and theoretical parameters of the axial interplanar spacings, lattice mismatches to WZ GaAs core, and strains in Pb_1−*x*_Sn_*x*_Te shells for different values of *x*.

	Fig. no	*d* [Å]		Axial mismatch *f* [%]		Dislocation distance [Å]		Axial strain *ε* [%]
		Theor	Exp. ± 0.05 Å	Theor	Exp	Theor	Exp	
Full-shell								
SnTe	2(c)	3.159	3.17	3.989	3.63	82.13	83 ± 7	0.348
Pb_0.65_Sn_0.35_Te	3(a)	3.206	3.22	2.464	2.02	131.40	187 ± 22	0.437
PbTe	5(b)	3.230	3.25	1.703	1.08	193.80	202 ± 23	0.619
Core								
wz-GaAs	–	${d}_{(0002)}^{{\text{exp}}={\text{theor}}}=3.285$ Å		–	–	–	–	–

#### Pb_0.44_Sn_0.56_Te half-shells

**Figure 7:**
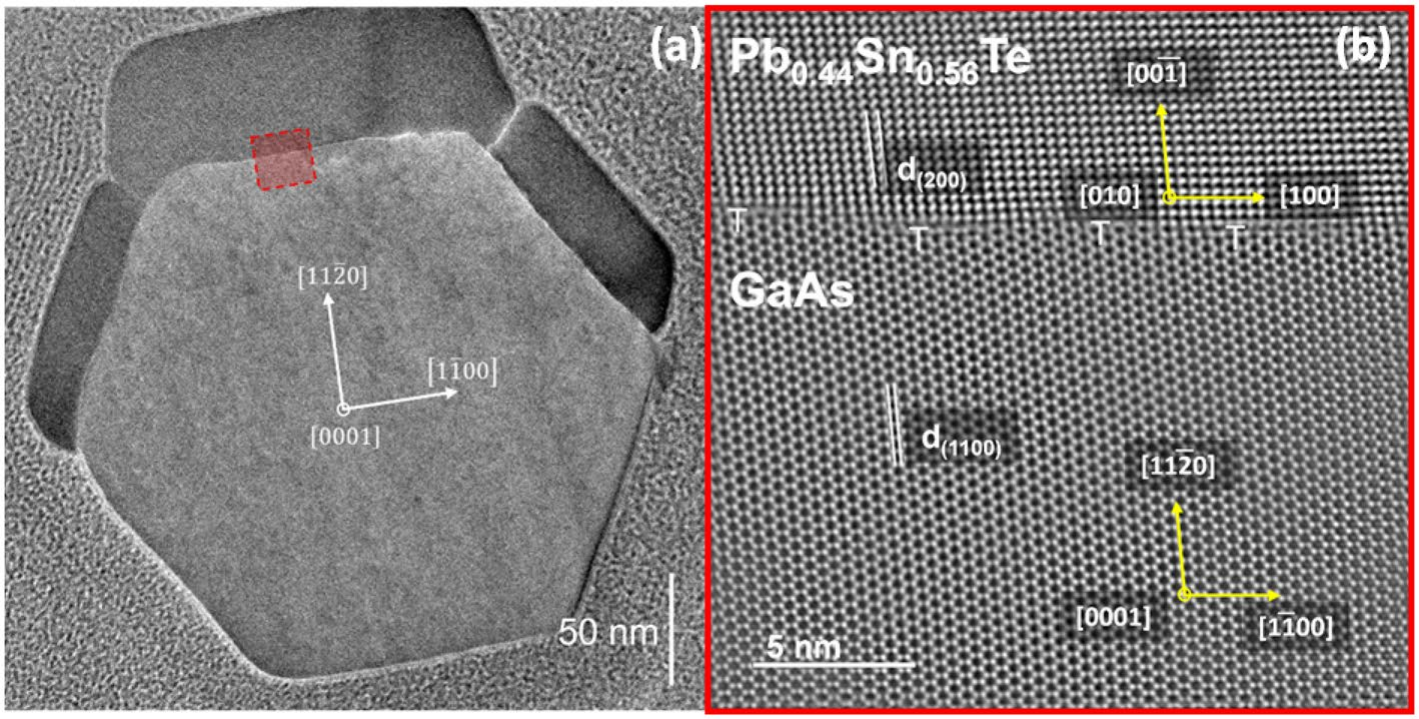
TEM images of GaAs_(core)_-Pb_0.44_Sn_0.56_Te_(half-shell)_ NW cross-section: (**a**) low-resolution image of the entire cross-section, (**b**) high-resolution image of the core–shell interface taken at the region marked with a red rectangle in panel (**a**). Misfit dislocations in the core–shell interface are marked with the single white “T” symbols.

The core-(half-shells) WZ GaAs/Pb_1−*x*_Sn_*x*_Te NWs (for *x* = 0.56) are grown without substrate rotation. The half-shell shown in Fig. 7a has the same crystalline perfection as the GaAs NW core, as evidenced in the high-resolution TEM image (Fig. 7b). The theoretical lattice mismatch along the NW axis is calculated using the interlayer spacing parameters ${d}_{(0002)}^{{\text{GaAs}}}=3.285$ Å and ${d}_{(020)}^{{\text{PbSnTe}}}=3.194$ Å. The value of this mismatch is ${f}_{{\text{theor}}\,[0001]}^{{\text{PbSnTe}}/{\text{GaAs}}}=0.0285.$ The lattice mismatch in the tangential direction (along the $[1\overline{1}00]$ azimuth of the GaAs NW core ), can be evaluated from the cross-sectional high-resolution TEM image. The measured spacing of Pb_0.44_Sn_0.56_Te (200) planes is ${d}_{(200)}^{{\text{PbSnTe}}}=3.22$ Å. Since we take reference ${d}_{(1\overline{1 }00)}^{{\text{GaAs}}}=3.451$ Å the measured mismatch between Pb_0.44_Sn_0.56_Te(200) and GaAs $\left(1\overline{1}00\right)$ is equal to ${f}_{{\text{exp}}\,[1\overline{1 }00]}^{{\text{PbSnTe}}/{\text{GaAs}}}=0.0717$ and the theoretical mismatch value calculated using bulk lattice parameters is ${f}_{{\text{theor}}\,[1\overline{1 }00]}^{{\text{PbSnTe}}/{\text{GaAs}}}=0.0805$. The interplanar spacing of the unstrained Pb_0.44_Sn_0.56_Te is ${d}_{(200)}^{{\text{PbSnTe}}}=3.194$ Å, which means that the shell is strained along the tangential $[1\overline{1}00]$ direction of the core and strain is ${\varepsilon }_{200}^{{\text{PbSnTe}}}\cong$ 0.814%. The calculated and measured tangential mismatch and strain values are summarised in Tab. 2.

**Table 2:** Experimental and theoretical parameters of the tangential interplanar spacings, lattice mismatch to WZ GaAs core and strain in Pb_0.44_Sn_0.56_Te half-shells.

		Fig. no.	d_(200)_ [Å]		Tangential mismatch f [%]		Dislocation distance [Å]		Tangential strain ε [%]
			Theor	Exp. ± 0.05 Å	Theor	Exp	Theor	Exp	
Half-shell	Pb_0.44_Sn_0.56_Te	7(b)	$3.194$	$3.22$	$8.05$	$7.17$	$37.8$	$53\pm 14$	$0.814$
Core	wz-GaAs		${d}_{(1\overline{1}00)}^{{\text{exp}}={\text{theor}}}=3.451$ Å						

We have demonstrated that IV–VI shells deposited on WZ GaAs NW sidewalls are under inhomogeneous tensile strain. It was reported that such strain can profoundly alter the topological surface states of IV–VI TCI^65,66^. Tensile strain can shift Dirac points towards X point of the surface Brillouin zone, whereas compressive strain can induce shift from trivial to topological regime e.g. in PbTe. Strain can be changed by tuning lattice parameters of WZ III–V NWs using e.g. (In,Ga)As or Ga(As,P) solid solutions as the NW cores, hence (III–V)/(IV–VI) core shell NWs are interesting also in this aspect.

## Conclusions

In summary, we have shown that PbTe, SnTe, and Pb_1−*x*_Sn_*x*_Te narrow bandgap semiconductors can be grown as full- and half-shells on the sidewalls of WZ GaAs NWs. Contrary to the uniform IV–VI NWs, this kind of hybrid NWs are easier to grow with larger lengths (determined by the III–V core NWs) which is beneficial for further studies of charge carriers transport involving topologically protected surface states occurring at {100} and {110} surfaces of Pb_1−*x*_Sn_*x*_Te. Among the contenders, the PbTe and Pb_0.65_Sn_0.35_Te full shells are best lattice-matched to the $\{11\overline{2}0\}$ sidewalls of WZ GaAs NWs, and the lattice mismatch along the [0001] core NW axis is 1.7% and 2.46%, respectively . The occurrence of the two types of sidewalls in WZ GaAs NWs enables the selection of orientation of IV–VI NW shells—the {100} IV–VI planes are best fitted to {$11\overline{2}0\}$ WZ GaAs NW sidewalls, whereas the {110} IV–VI planes are fitted to the $\{1\overline{1}00\}$ WZ GaAs ones. This latter opens possibilities for investigations of topological {110} surfaces of IV–VI TCI which have not been studied so far. To our best knowledge, the planar growth of narrow bandgap IV–VI in (110) orientation has not been reported yet, and theoretically predicted topological states on this surface are still to be experimentally confirmed.

## Methods

### Molecular beam epitaxy (MBE)

In the first step we have grown Au-catalysed GaAs NWs on GaAs (111)B substrates in the III–V MBE system. The NWs have been grown in conditions favouring wurtzite (WZ) crystal structure, i.e. with a high As/Ga flux ratio^67^. They grow perpendicularly to the substrate surface, in [0001] direction. Then, III–V NWs were transferred to the separate IV–VI MBE system through ambient air and used as templates (cores) for IV–VI shells deposition. Prior to the deposition of IV–VI shells, GaAs NWs were annealed at about 590 °C to thermally desorb the native oxides from the NW sidewalls. The annealing temperature has been calibrated by observations of thermal desorption of oxides from planar GaAs substrates deduced from reflection high energy electron diffraction (RHEED) images. Even though we cannot directly observe the oxide desorption from NW sidewalls based on RHEED images (in this case due to transmission of e-beam through NWs), we infer that the sidewalls of WZ GaAs NWs are smooth even if the annealing temperature was slightly higher. This is based on the results of Paul Schmiedeke et al*.* who investigated in-situ annealing of WZ GaAs NWs (also see S.1.1 in Supplementary information)^68^.

After annealing, the substrate temperature was decreased down to (400 °C < *T* < 450 °C) to start the growth of the IV–VI shells. For calibration of growth temperatures usually we use two methods: melting points of known materials – in our case In, Sn, and Pb, as well as desorption of oxides from GaAs(111)B, the second method relies on a pyrometer. The melting points are measured with uncertainty of about 5 °C. The pyrometer was used mainly to find the dependence between the temperature of the heater (measured using thermocouple coupled to the heater) and the substrate, and next it was corrected by relation taken from the melting points estimation. In general temperatures in the MBE system are not easy to measure exactly due to other factors, e.g., way of mounting the substrate to the holder. Full-shells are grown with relatively slow substrate rotation of 6 degrees per second, whereas the half-shells are grown in static conditions, i.e. without substrate rotation (see Fig. S3 in Supplementary information). The latter enables the deposition of material on three out of six core NW sidewalls only. Pb_1−*x*_Sn_*x*_Te shells with various chemical compositions are grown with *x* ranging from 0 to 1. Calibrations of the chemical composition of Pb_1−*x*_Sn_*x*_Te solid solution shells are based on the data obtained for the planar growth. For the specific Sn content in Pb_1−*x*_Sn_*x*_Te shells, the same molecular flux intensities were used as those applied in the planar growth. Compositions of the layers were calibrated using energy-dispersive X-ray spectroscopy. See the relevant calibration curve in the Supplementary information (Fig. S1). Time of the growth for the deposition of shells was maintained to achieve the required thickness of a deposited material (see Fig. S2 in Supplementary information).

### High resolution transmission electron microscope

The structure of GaAs/Pb_1−*x*_Sn_*x*_Te core–shell nanowires was investigated using FEI Titan 80–300 transmission electron microscope operating at 300 kV with an aberration correction in HRTEM. Typical examples of NWs presented in the paper are representative and similar to other TEM specimens. Nanowires were analysed after deposition on a thin carbon film spread over a copper lace and dry transferred mechanically from the substrate; their orientation on a carbon film was random. During TEM analysis, in order to produce good quality images, the observed structure needs to be rotated toward desired orientation and we cannot state, which or if any orientation was preferred during the transfer of NWs from the substrate to a carbon film. Perpendicular cross-section of GaAs_(core)_/Pb_1−*x*_Sn_*x*_Te_(half-shell)_ nanowire was prepared using focused gallium ion beam in Helios nanolab 600 focused ion beam (FIB). During cutting process, the surface of nanowire was protected by electron-deposited Pt − C composite from a metalorganic source. The scanning transmission electron microscopy high angle annular dark field detector (STEM − HAADF) images were acquired at the scattering angle range between 80 mrad and 200 mrad camera length, with a converged semi-angle of 9.5 mrad of the incident beam.

### Supplementary Information

Supplementary Information.

## Data Availability

The datasets used and analysed during the current study are available from the corresponding author on a reasonable request.

## References

[1] Hull R, Bean JC (1992). Misfit dislocations in lattice-mismatched epitaxial films. Crit. Rev. Solid State Mater. Sci..

[2] Jain S, Harker A, Cowley R (1997). Misfit strain and misfit dislocations in lattice mismatched epitaxial layers and other systems. Philos. Mag. A.

[3] Kunert B (2018). How to control defect formation in monolithic III/V hetero-epitaxy on (100) Si? A critical review on current approaches. Semicond. Sci. Technol..

[4] Chandra Y, Flores ES, Adhikari S (2020). Buckling of 2D nano hetero-structures with moire patterns. Comput. Mater. Sci..

[5] Sugumaran PJ, Zhang J, Zhang Y (2022). Synthesis of stable core-shell perovskite based nano-heterostructures. J. Colloid Interface Sci..

[6] Hasan MZ, Kane CL (2010). Colloquium: Topological insulators. Rev. Mod. Phys..

[7] Qi XL, Zhang SC (2011). Topological insulators and superconductors. Rev. Mod. Phys..

[8] Springholz G (2018). Molecular Beam Epitaxy.

[9] Liu H (2020). Photothermoelectric SnTe photodetector with broad spectral response and high on/off ratio. ACS Appl. Mater. Interfaces.

[10] Safaei S, Kacman P, Buczko R (2013). Topological crystalline insulator (Pb, Sn) Te: Surface states and their spin polarization. Phys. Rev. B.

[11] Fu L (2011). Topological crystalline insulators. Phys. Rev. Lett..

[12] Hsieh TH (2012). Topological crystalline insulators in the SnTe material class. Nat. Commun..

[13] Tanaka Y (2012). Experimental realization of a topological crystalline insulator in SnTe. Nat. Phys..

[14] Xu S-Y (2012). Observation of a topological crystalline insulator phase and topological phase transition in Pb_1−x_Sn_x_Te. Nat. Commun..

[15] Dziawa P (2012). Topological crystalline insulator states in Pb_1−x_Sn_x_Se. Nat. Mater..

[16] Krizman G (2018). Tunable Dirac interface states in topological superlattices. Phys. Rev. B.

[17] Assaf B (2016). Massive and massless Dirac fermions in Pb_1−x_Sn_x_Te topological crystalline insulator probed by magneto-optical absorption. Sci. Rep..

[18] Schindler F (2018). Higher-order topological insulators. Sci. Adv..

[19] Schindler F (2018). Higher-order topology in bismuth. Nat. Phys..

[20] Wu Y (2004). Controlled growth and structures of molecular-scale silicon nanowires. Nano Lett..

[21] Dubrovskii V (2005). Diffusion-induced growth of GaAs nanowhiskers during molecular beam epitaxy: Theory and experiment. Phys. Rev. B.

[22] Janik E (2006). ZnTe nanowires grown on GaAs (100) substrates by molecular beam epitaxy. Appl. Phys. Lett..

[23] Spirkoska D (2009). Structural and optical properties of high quality zinc-blende/wurtzite GaAs nanowire heterostructures. Phys. Rev. B.

[24] Bauer B (2010). Position controlled self-catalyzed growth of GaAs nanowires by molecular beam epitaxy. Nanotechnology.

[25] Maliakkal CB (2019). In situ analysis of catalyst composition during gold catalyzed GaAs nanowire growth. Nat. Commun..

[26] Wagner AR, Ellis SW (1964). Vapor-liquid-solid mechanism of single crystal growth. Appl. Phys. Lett..

[27] Dziawa P (2010). Defect free PbTe nanowires grown by molecular beam epitaxy on GaAs (111) B substrates. Cryst. Growth Des..

[28] Panciera F (2020). Phase selection in self-catalyzed GaAs nanowires. Nano Lett..

[29] Parameshwaran V, Taylor P (2019). Alloying Behavior and Crystallinity of (111)-Oriented Lead Tin Telluride Grown on (100)-Oriented Gallium Arsenide.

[30] Yang L (2010). Novel route to scalable synthesis of II–VI semiconductor nanowires: Catalyst-assisted vacuum thermal evaporation. J. Cryst. Growth.

[31] Dick KA (2008). A review of nanowire growth promoted by alloys and non-alloying elements with emphasis on Au-assisted III–V nanowires. Prog. Cryst. Growth Charact. Mater..

[32] Wang J (2013). Core/shell colloidal quantum dot exciplex states for the development of highly efficient quantum-dot-sensitized solar cells. J. Am. Chem. Soc..

[33] Reiss P, Protiere M, Li L (2009). Core/shell semiconductor nanocrystals. Small.

[34] Gao PX, Lao CS, Ding Y, Wang ZL (2006). Metal/semiconductor core/shell nanodisks and nanotubes. Adv. Funct. Mater..

[35] Brumer M (2005). PbSe/PbS and PbSe/PbSe_x_S_1–__x_core/shell nanocrystals. Adv. Funct. Mater..

[36] Kockert M (2021). Semimetal to semiconductor transition in Bi/TiO 2 core/shell nanowires. Nanoscale Adv..

[37] Tang X (2019). Single halide perovskite/semiconductor core/shell quantum dots with ultrastability and nonblinking properties. Adv. Sci..

[38] Sköld N (2005). Growth and optical properties of strained GaAs−Ga_x_In_1__−__x_P core−shell nanowires. Nano Lett..

[39] Cirloganu CM (2014). Enhanced carrier multiplication in engineered quasi-type-II quantum dots. Nat. Commun..

[40] Miranti R (2020). Exclusive electron transport in Core@ Shell PbTe@ PbS colloidal semiconductor nanocrystal assemblies. ACS Nano.

[41] Aryal, S. & Pati, R. PbTe (core)/PbS (shell) Nanowire: Electronic structure, thermodynamic stability, and mechanical and optical properties. *J. Phys. Chem. C* (2021).

[42] Zhang H, Man B, Zhang Q (2017). Topological crystalline insulator SnTe/Si vertical heterostructure photodetectors for high-performance near-infrared detection. ACS Appl. Mater. Interfaces.

[43] Ginting D (2018). Enhancement of thermoelectric performance in Na-doped Pb_0.6_Sn_0.4_Te_0.95_–xSexS_0.05_ via breaking the inversion symmetry, band convergence, and nanostructuring by multiple elements doping. ACS Appl. Mater. Interfaces.

[44] Haidet BB (2021). Interface structure and luminescence properties of epitaxial PbSe films on InAs (111) A. J. Vac. Sci. Technol. A Vac. Surf. Films.

[45] Sadowski J, Herman M (1995). Hard heteroepitaxy of molecular beam epitaxial grown PbTe on off oriented GaAs (100) substrates. J. Cryst. Growth.

[46] Liu X (2021). Unraveling the structural and electronic properties of strained PbSe on GaAs. J. Cryst. Growth.

[47] Yan X, Fan S, Zhang X, Ren X (2015). Analysis of critical dimensions for nanowire core-multishell heterostructures. Nanoscale Res. Lett..

[48] Pollard K, Erbil A, Sudharsanan R, Perkowitz S (1992). Metalorganic chemical vapor deposition of PbTe films on GaAs substrates. J. Appl. Phys..

[49] Sulich A (2022). Unit cell distortion and surface morphology diversification in a SnTe/CdTe (001) topological crystalline insulator heterostructure: Influence of defect azimuthal distribution. J. Mater. Chem. C.

[50] Sadowski J (2018). Defect-free SnTe topological crystalline insulator nanowires grown by molecular beam epitaxy on graphene. Nanoscale.

[51] Nguyen NM, Brzezicki W, Hyart T (2022). Corner states, hinge states, and Majorana modes in SnTe nanowires. Phys. Rev. B.

[52] Ketterer B, Heiss M, Uccelli E, Arbiol J, FontcubertaiMorral A (2011). Untangling the electronic band structure of wurtzite GaAs nanowires by resonant Raman spectroscopy. ACS Nano.

[53] Liu J, Qian X, Fu L (2015). Crystal field effect induced topological crystalline insulators in monolayer IV–VI semiconductors. Nano Lett..

[54] Liu J (2014). Spin-filtered edge states with an electrically tunable gap in a two-dimensional topological crystalline insulator. Nat. Mater..

[55] Sessi P (2016). Robust spin-polarized midgap states at step edges of topological crystalline insulators. Science.

[56] Jacobsson D (2015). Phase transformation in radially merged wurtzite GaAs nanowires. Cryst. Growth Des..

[57] Bauer Pereira P (2013). Lattice dynamics and structure of GeTe, SnTe and PbTe. Physica Status Solidi B.

[58] Ferrand D, Cibert J (2014). Strain in crystalline core-shell nanowires. Eur. Phys. J. Appl. Phys..

[59] Johnston WD, King JG (1966). Measurement of velocity distributions of atoms evaporating from liquid helium ii. Phys. Rev. Lett..

[60] Preier H (1979). Recent advances in lead-chalcogenide diode lasers. Appl. Phys..

[61] Volobuev VV (2017). Giant Rashba splitting in Pb_1−x_Sn_x_Te (111) topological crystalline insulator films controlled by Bi doping in the bulk. Adv. Mater..

[62] Wagner JW, Woolley JC (1967). Phase studies of the Pb_1−x_Sn_x_Te alloys. Mater. Res. Bull..

[63] Barone P (2013). Pressure-induced topological phase transitions in rocksalt chalcogenides. Phys. Rev. B.

[64] Rajaji V, Manjón F, Narayana C (2022). Pressure induced topological and topological crystalline insulators. J. Phys. Condens. Matter.

[65] Zeljkovic I (2015). Strain engineering Dirac surface states in heteroepitaxial topological crystalline insulator thin films. Nat. Nanotechnol..

[66] Walkup D (2018). Interplay of orbital effects and nanoscale strain in topological crystalline insulators. Nat. Commun..

[67] Dheeraj D (2012). Controlling crystal phases in GaAs nanowires grown by Au-assisted molecular beam epitaxy. Nanotechnology.

[68] Schmiedeke P, Panciera F, Harmand J-C, Travers L, Koblmüller G (2023). Real-time thermal decomposition kinetics of GaAs nanowires and their crystal polytypes on the atomic scale. Nanoscale Adv..

