# Supplementary material for: Axially lattice-matched wurtzite/rock-salt GaAs/Pb1−xSnxTe nanowires
Source: Sci Rep. 2024 Jan 5;14:589. doi: 10.1038/s41598-024-51200-w (PMC10770406; doi:10.1038/s41598-024-51200-w)
Supplement: Supplementary file 1 — Supplementary Information. [file 41598_2024_51200_MOESM1_ESM.docx]

**Supplementary Information**

**Axially lattice-matched wurtzite/rock-salt**

**GaAs/Pb_1-_*_x_*Sn*_x_*Te nanowires**

**Sania Dad^1^, Piotr Dziawa^1^, Wiktoria Zajkowska-Pietrzak^1^, Sławomir Kret^1^, Mirosław Kozłowski^1^, Maciej Wójcik^1^, and Janusz Sadowski^1,2,3^**

^1^Institute of Physics, Polish Academy of Sciences, Aleja Lotnikow 32/46, PL-02-668 Warsaw, Poland

^2^Faculty of Physics, University of Warsaw, Pasteura 5, PL- 02093 Warsaw, Poland

^3^Ensemble3 Centre of Excellence, Wolczynska Str. 133, PL-01-919 Warsaw, Poland

**S.1. METHODS**

**S.1.1 Oxides desorption form the sidewalls of NWs:**

Typically surfaces of planar samples grown in the MBE system or the sidewalls of NWs are protected by amorphous layer of material with lower vapour pressure if transferring between different vacuum systems under ambient pressure. For example GaAs NWs capped by amorphous As were reported e.g. by *X. Guan, et al., Nanoscale,* ***8****, 15637 (2016)*. Although based on our experience with thermal annealing of uncapped wurtzite (WZ) GaAs NWs we concluded that As capping is not necessary in this case. The MBE substrate heater thermocouple was calibrated through RHEED observations of native oxide desorption from planar GaAs(100) substrates.

WZ GaAs {11-20} equivalent to zinc blende (ZB) GaAs {110} is a stable nonpolar surface. Careful desorption of the surface oxide from commercial epi-ready GaAs(100) even without impinging As flux results in a smooth surface with streaky RHEED patterns. Evidently WZ GaAs substrates are not available since GaAs bulk does not occur in the WZ phase so this cannot be tested this way. However vacuum annealing of both WZ and ZB GaAs NWs was studied in-situ in TEM and reported by *Paul Schmiedeke, Federico Panciera, Jean-Christophe Harmand, Laurent Travers and Gregor Koblmüller*, *Nanoscale Adv.,* ***5****, 2994 (2023)*. Even though the NWs studied by the authors have also been grown in-situ in TEM vacuum system (so the NW facets were not oxidized), the authors conclude that in WZ GaAs NWs overheated to initiate the GaAs desorption from the sidewalls the desorption proceeds by a monolayer step flow mode and does not induce the sidewall surface roughening, in contrast to the case of ZB GaAs NWs. Hence we conclude that even slight overheating of WZ NWs above the oxide desorption temperature will not induce the sidewalls roughening. The effect or role of gold catalyst droplet during sidewall oxide desorption can be neglected. The droplet is localised at the NW top and does not slide down towards the NW base during thermal treatment.

**S.1.2. Chemical analysis of 2D Pb_1-_*_x_*Sn*_x_*Te:**

We have grown series of Pb_1-_*_x_*Sn*_x_*Te layers in our home-built MBE system. The chemical composition analysis was carried out using energy dispersive X-ray (EDX) spectrometer (Oxford Instruments), given in Supplementary Fig. S1. The analysis was performed at an accelerating voltage of 15 kV.


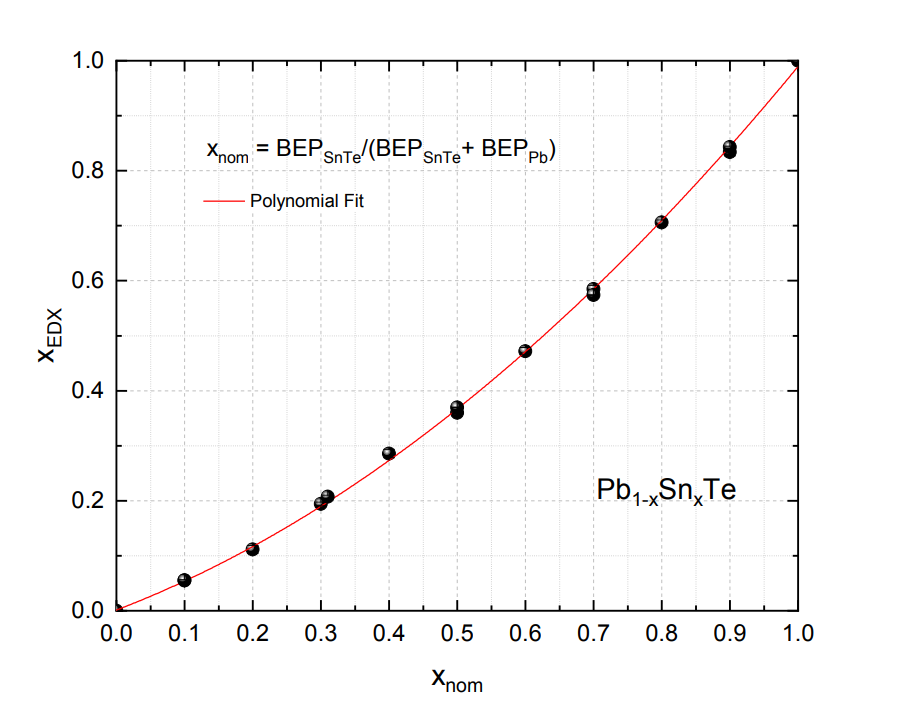


**Figure S1:** Experimental data points for *x*-EDX vs. *x*-nominal (nom)

**S.1.3. Rate of growth in our IV-VI MBE system:**

The growth times and fluxes were calibrated to attain a controlled growth rate ranging from 0.1 Å/s to 0.5 Å/s equivalent 2D growth. We use the calibration data for planar growth, which are shown in Supplementary Fig S2. The growth rate on NW shells was obtained via geometrical factor corrections taking into account the angle between the effusion cells axes and normal to the substrate heater plane, which also defines the NW axes direction if core NW are perpendicular to the substrate on which they are grown {GaAs(111)B in our case}. The chemical composition of the ternary compounds was estimated from calibrations of the molecular fluxes for the layers to obtain *x* = 0.5 with an accuracy less than 0.01.


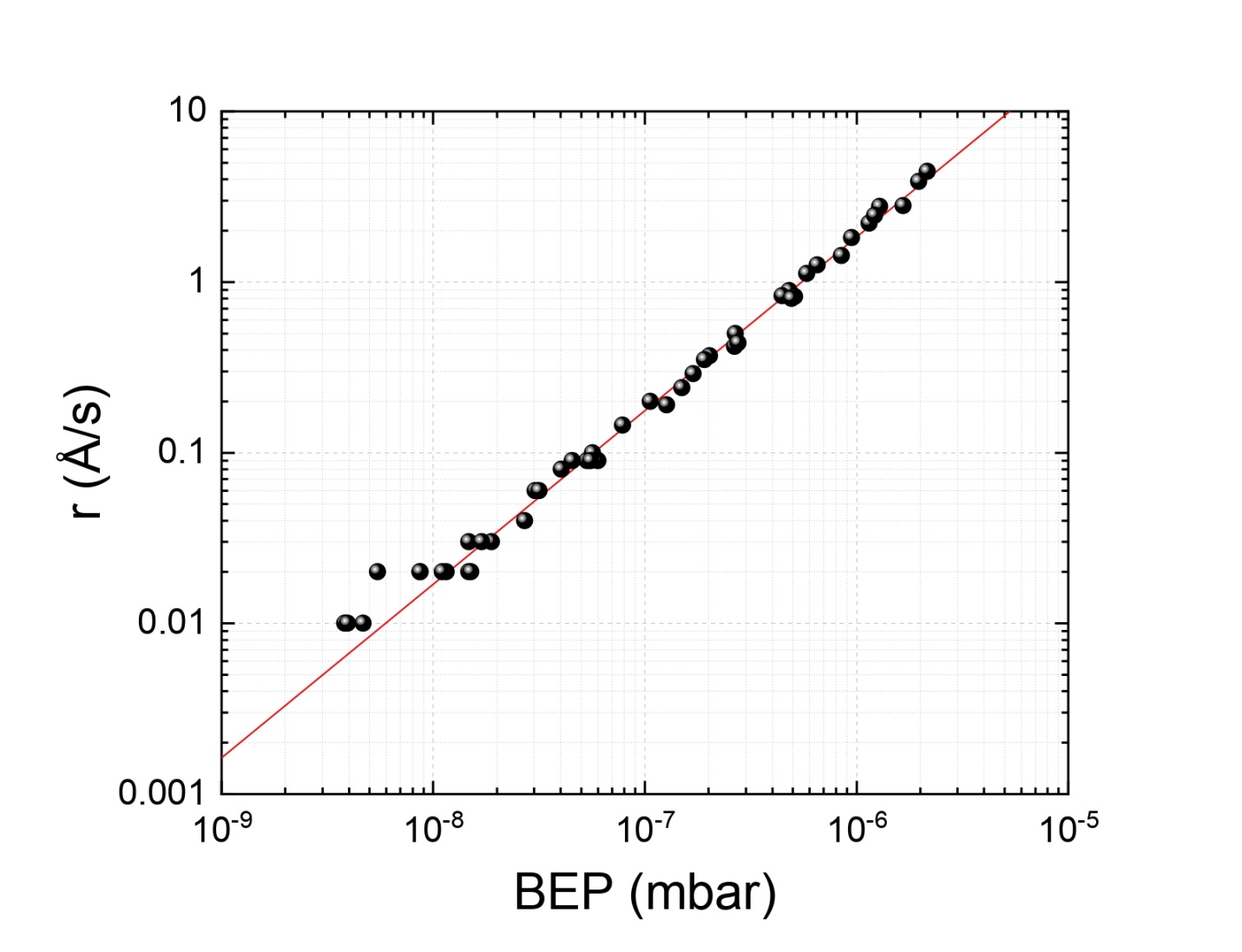


**Fig. S2:** Experimental data of the rate of growth (*r*) vs. beam equivalent pressure (BEP) for all of the sources mounted in our MBE system.

**S.1.4. Possible orientation of NWs during growth of half-shells:**

Half-shells of Pb_1-_*_x_*Sn*_x_*Te by fixing the substrates to the holder (glued to Mo-blocks by liquid InGa mixture) at a specific angle determined by the known edge direction of piece cut from GaAs wafer which is always parallel to <-110> crystallographic direction (corresponding to the GaAs easy cleavage plane). The GaAs NWs templates are oriented azimuthally with respect to the orientation of the substrate crystallographic directions. Hence, in each deposition process, we maintained almost the same orientation of the NWs (angle uncertainties are less than 5 degrees) with respect to the fixed geometrical position of the sources in the growth chamber. Anyway, surface mobility of the adatoms results in coverage of sidewalls exposed to the molecular fluxes (see the cross-section of the half-shell in Fig. 7 in the main article file).

If one assumed a different orientation of the NWs relative to the (averaged) flux, e.g. case (B), then a different coverage of the core might be possible (see Supplementary Fig. S3). However, we have not carried out such experiment.

**
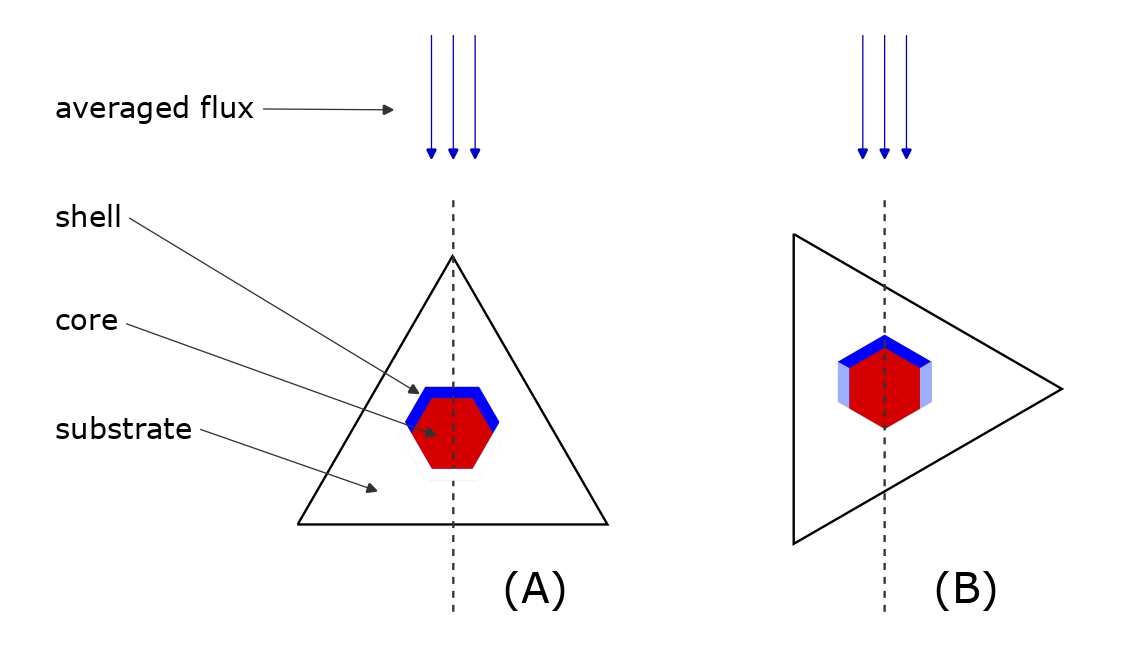
**

**Figure S3:** Schematic cross-sectional image of NWs depicting the possible two distinct orientations during growth of half-shell. Here the big triangles denotes the substrate (easy cleavage plane of (111) GaAs). Here, red and blue color depicts core and shell of NWs, respectively.

**S.2. Origin of moire fringes:**

Moiré patterns or moiré fringes are formed when two set of line interfere with a periodicity. There are two main types for such patterns that is; translational moiré and rotational moiré. The translational contribution is arising from the difference in lattice constants between the core and shell fragments and rotational effects results from the relative rotation between them. However in general, the rotation between core and shell grain can vary *(D. B. Williams, C. B. Carter; Transmission Electron Microscopy-A Textbook for Material Science* *Microscopy and Microanalysis, Volume 5, Issue 6, 1999.*

Translational moiré fringes eq: $d_{tm}=\frac{1}{g_{tm}}=\frac{1}{g_{2}-g_{1}}=\frac{d_{2}d_{1}}{d_{1}-d_{2}}$

Rotational moiré fringes eq: $d_{rm}=\frac{1}{2gsin\left( \frac{\beta}{2} \right)}=\frac{d}{2\sin\left( \frac{\beta}{2} \right)}$

General moiré fringes eq: $d_{gm}=\frac{d_{1}d_{2}}{\left( \left( d_{1}-d_{2} \right)^{2}+d_{1}d_{2}\beta^{2} \right)^{0.5}}$

Figure S4 shows the GaAs-(Pb,Sn)Te core-shell NW with well visible moiré fringes in both axial and tangential direction. Large difference in the lattice mismatch in axial and tangential direction (perpendicular to the NW axis and parallel to the sidewall) is reflected by much different periods of moiré fringes: 16.94 nm and 4.86 nm, for axial and tangential fringes, respectively.


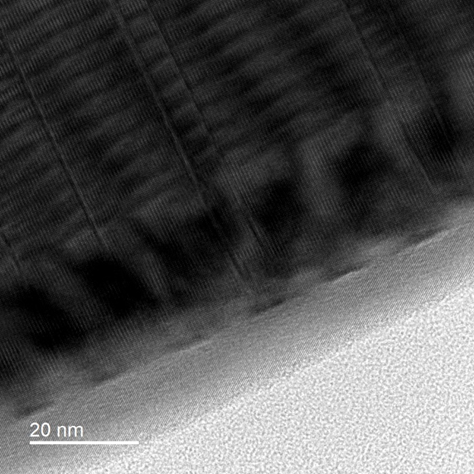

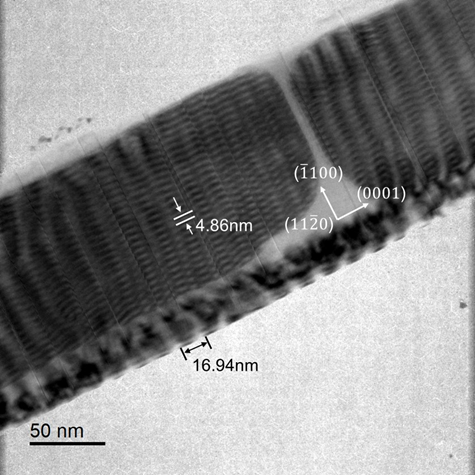


**Figure S4.** Moiré fringes of WZ GaAs nanowire with Pb_0.47_Sn_0.53_Te shell. Two kinds of moiré fringes are visible: long period fringes along the NW axis with 16.94 nm average distance between them and short period fringes perpendicular to the NW axis with 4.86 nm average distance. This visualizes large differences in lattice mismatches between the core and the shell in these two directions.

As shown in Supplementary Fig. S4 there is a gap in the Pb_1-_*_x_*Sn*_x_*Te shell, seen along the NW axis. In general such gaps are quite common in our WZ GaAs – Pb_1-_*_x_*Sn*_x_*Te core-shell NWs for shell thickness up to about 10 nm. The moiré fringes can be studied in more depth but such study is out of scope of this article.

**S.3. Schematic of NW**

A model image of a NW with noncontinuous shell is shown in Supplementary Fig. S5. This diagram explains the darker and lighter areas seen in Figure 5a (in the main text) resulting from the projection of the shell from both sides.


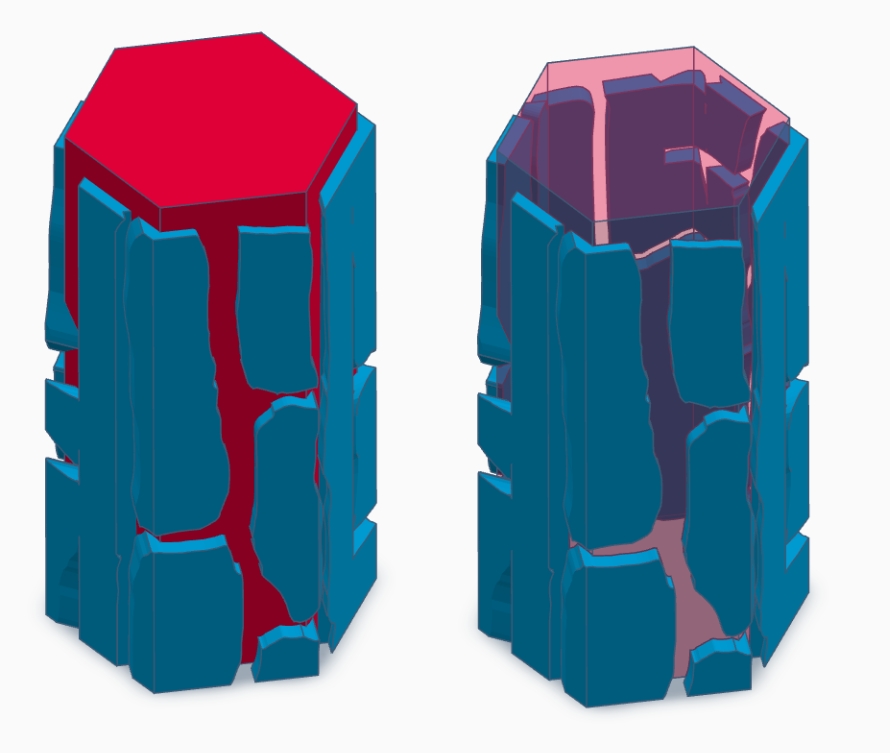


**Fig. S5.** Schematic representation of a core-shell structure of WZ NW with a discontinuous shell. The core is transparent to reveal the back side of the NW.
